# Complete Genome Sequence of *Tissierella* sp. Strain Yu-01, Isolated from the Feces of the Black Soldier Fly

**DOI:** 10.1128/mra.00277-23

**Published:** 2023-05-08

**Authors:** Junyu Zhu, Guowen Dong, Chao-Jen Shih, Yen-Chi Wu, Shu-Jung Lai, Yi-Ting You, Wanling Qiu, Chih-Hung Wu, Ching-Hua Liao, Yan Gong, Sheng-Chung Chen

**Affiliations:** a College of Environment and Safety Engineering, Fuzhou University, Fuzhou, Fujian, People’s Republic of China; b School of Resources and Chemical Engineering, Sanming University, Sanming City, Fujian, People’s Republic of China; c Bioresource Collection and Research Center, Food Industry Research and Development Institute, Hsinchu, Taiwan, Republic of China; d Graduate Institute of Biomedical Sciences, China Medical University, Taichung City, Taiwan, Republic of China; e Research Center for Cancer Biology, China Medical University, Taichung City, Taiwan, Republic of China; f Department of Life Sciences, National Chung Hsing University, Taichung City, Taiwan, Republic of China; g People’s Government of Zhongcun Township, Sanyuan District, Sanming City, Fujian, People’s Republic of China; University of Maryland School of Medicine

## Abstract

We report the complete genome sequence of *Tissierella* sp. strain Yu-01 (=BCRC 81391), isolated from the feces of black soldier fly (Hermetia illucens) larvae. This fly has increasingly been gaining attention because of its usefulness for recycling organic waste. The genome of strain Yu-01 was selected for further species delineation.

## ANNOUNCEMENT

Strains in the genus *Tissierella* are obligately anaerobic, rod-shaped, nonsporulating bacteria (1). The type species is Tissierella praeacuta (2, 3). The 7 characterized and described *Tissierella* species include *T. praeacuta* (2, 3), T. creatinine (4), T. creatinophila (5), T. carlieri (6), T. pigra (7), T. hominis (8), and T. simiarum (9). These strains were isolated from animal intestine or feces (2, 3, 7–9), human skin and soft tissue (6), or sewage sludge (4, 5). Here, we report a bacterium, designated Yu-01, isolated from the feces of larvae of the black soldier fly (Hermetia illucens), which has increasingly been gaining attention because of its usefulness for recycling organic waste (10). Black soldier fly eggs were purchased from a shop in Taobao in June 2020 and then farmed by a colleague at our university. A feces sample was obtained on 30 May 2021. The strain was isolated using the serial dilution method and the roll-tube technique three times. The genome of strain Yu-01 was selected for sequencing for species delineation and comparative genomic analysis.

Strain Yu-01 was deposited at the Bioresource Collection and Research Center, Taiwan, as strain BCRC 81391. It was grown in an anaerobic MB/W medium and incubated at 28°C, according to the methods described in our previous publications (11–13). Genomic DNA of strain Yu-01 was extracted using the NucleoBond high-molecular-weight (HMW) DNA kit (Macherey-Nagel, Germany) according to the manufacturer’s instruction. The genome was sequenced at Sangon Biotech (Shanghai) Co., Ltd., using the DNBSEQ-T7 platform (MGI Tech Co., Ltd.).

Genomic DNA was sheared randomly, and a DNA library (paired-end, 150-bp [PE150] format) was constructed using the Hieff next-generation sequencing (NGS) MaxUp II DNA library prep kit for Illumina (Yeasen Biotechnology [Shanghai] Co., Ltd.). The constructed library was sequenced using the MGISEQ-2000RS high-throughput sequencing set (PE150 format), and 6,541,912 reads were generated. All generated reads were quality trimmed to obtain high-quality reads using Trimmomatic v0.36 (14). These reads were *de novo* assembled using SPAdes v3.10.1 (15), and the quality of the assembled genome was evaluated using QUAST v4.5 (16). The sequencing protocol generated ~333× mean coverage of the genome. The assembly generated a single large contig of 2,947,135 bp, which was circularized by aligning both ends of the contig sequences and deleting the overlapping sequences of one end. The genes of the genome were identified using the Prokaryotic Genome Annotation Pipeline (PGAP) at the website of the National Center for Biotechnology Information (http://www.ncbi.nlm.nih.gov) (17–19). Default parameters were used for all software unless otherwise specified.

The complete genome of strain Yu-01 comprised 2,947,135 bp and 32.23% GC content. The genome was predicted to have 2,976 genes, of which 2,877 were protein coding. The genome contains 21 rRNA genes and 62 tRNA genes. Two clustered regularly interspaced short palindromic repeats (CRISPRs) with a high evidence level were found in the genome using CRISPRCasFinder (20). Default parameters were used for all bioinformatics analyses.

### Data availability.

The genome sequence of strain Yu-01 has been deposited at GenBank under accession number CP120677. The version described in this paper is the first version. The BioProject and BioSample accession numbers are PRJNA946127 and SAMN33811732, respectively. The raw sequence reads were deposited in the Sequence Read Archive (SRA) under accession number SRR23924617.
